# Prevalence of amenorrhea in elite female competitive climbers

**DOI:** 10.3389/fspor.2022.895588

**Published:** 2022-08-10

**Authors:** Lanae Joubert, Amity Warme, Abigail Larson, Gudmund Grønhaug, Marisa Michael, Volker Schöffl, Eugen Burtscher, Nanna Meyer

**Affiliations:** ^1^School of Health and Human Performance, Northern Michigan University, Marquette, MI, United States; ^2^Department of Human Physiology and Nutrition, William J. Hybl Sports Medicine and Performance Center, University of Colorado Colorado Springs, Colorado Springs, CO, United States; ^3^Department of Kinesiology and Outdoor Recreation, Southern Utah University, Cedar City, UT, United States; ^4^Department of Sport, Food and Natural Sciences Faculty of Education, Arts and Sports, Western Norway University of Applied Sciences, Campus Sogndal, Norway; ^5^Real Nutrition, LLC, Portland, OR, United States; ^6^Department of Orthopedic and Trauma Surgery, Klinikum Bamberg, Bamberg, Germany; ^7^School of Clinical and Applied Sciences, Leeds Becket University, Leeds, United Kingdom; ^8^Department of Orthopedic and Trauma Surgery, Friedrich Alexander University Erlangen-Nuremberg, Erlangen, Germany; ^9^Section of Wilderness Medicine, Department of Emergency Medicine, University of Colorado Boulder, Boulder, CO, United States; ^10^Medical Commission of the International Federation of Sport Climbing, Turino, Italy

**Keywords:** competition, rock, climbing, diet, sports medicine, menstrual dysfunction, low energy availability risk

## Abstract

Elite competitive sport climbers exhibit a high strength-to-weight ratio and are reported in the literature to be lighter and leaner than their athletic counterparts. Current research regarding nutrition among climbers is sparse but suggests that they may be at high risk for low energy availability and Relative Energy Deficiency in Sport (RED-S). The prevalence of amenorrhea, one of the primary indicators of RED-S, is unknown in this athletic population. The purpose of this study was to determine the prevalence of current (previous 12 months) amenorrhea among elite level competitive sport climbers.

**Methods:**

An anonymous online survey was distributed *via* email to 1,500 female climbers registered as competitors within the International Federation of Sport Climbing to assess the prevalence of amenorrhea over the past 12 months.

**Results:**

A total of 114 female sport climbers answered all survey questions regarding menstrual function and 18 athletes (15.8%) presented with current amenorrhea. The majority of the athletes (72%; *n* = 82) were categorized with eumenorrhea. An additional 14 athletes (12.3%) provided information that indicated irregular cycles, but answers to all menstrual cycle questions were not congruent to elicit a classification of amenorrhea and these athletes were categorized with a menstrual status of unsure. The average BMI for climbers with eumenorrhea was 20.8 ± 1.8 kg/m^2^ and 19.9 ± 2.4 kg/m^2^ for those with amenorrhea. A higher percentage of climbers with amenorrhea revealed they currently struggle with an eating disorder compared to those without amenorrhea (13.5 vs. 22.2%, respectively).

**Conclusion:**

This study indicates that some female climbers competing at the World Cup level do have menstrual disturbances with relatively normal BMIs and some currently struggle with one or more eating disorders. Even though World Cup competitions use BMI critical margins to screen competitors, this research highlights the need for more medical supervision of competitive elite female sport climbers in order to protect their overall health, including menstrual function. Further research is required to clarify how many climbers suffer from endocrine abnormalities related to RED-S. With more scientific evidence in this area practitioners will be better equipped to educate the athlete and coach with evidence-based nutrition recommendations.

## Introduction

Research has demonstrated that female athletes display a higher prevalence of menstrual disturbances than their non-athletic counterparts (De Souza and Williams, 2004; Beals and Hill, 2006; De Souza et al., 2010). Furthermore, athletes participating in lean-build sports where strength-to-weight ratio may be a performance attribute, such as in climbing, are at increased risk for menstrual disturbances due to a variety of reasons, one being low energy availability (LEA) with or without body weight changes. LEA is a condition in which energy intake is inadequate to cover all physiological functions after compensating for exercise energy expenditure (Mountjoy et al., 2018), which in athletes is most commonly referred to as Relative Energy Deficiency in Sport (RED-S). Climbers often strive to reach an optimal strength-to-weight ratio, which is a characteristic known to assist climber success (Saul et al., 2019). The pursuit to achieve this competitive attribute may place athletes at an increased risk for menstrual disturbances (Beals, 2002; Torstveit and Sundgot-Borgen, 2005; Nichols et al., 2007; Meng et al., 2020).

Adequate energy is required to maintain reproductive processes and data support that disruptions to the hypothalamic pituitary ovarian axis (HPO axis) leads to reproductive function suppression during conditions of chronic LEA (Estienne et al., 2021). The HPO axis helps to monitor and prioritize fuel for vital bodily processes such as heart and lung function and locomotion, and to conserve energy from current non-relevant functions such as gestation and lactation (De Souza and Williams, 2004; Mountjoy et al., 2018; Meng et al., 2020; Estienne et al., 2021). Therefore, it is likely that menstrual disturbances may occur in athletes training with RED-S.

Previous studies have examined the prevalence of menstrual disturbances in a variety of athletes as presented in Table 1. The criteria for defining amenorrhea and oligomenorrhea vary between studies, but 7 of the 9 studies define secondary amenorrhea by the same definition used in the current study: *absence of menstrual bleeding for at least three consecutive months or fewer than 4 cycles in the past year* (Wade et al., 1996; Goodman and Warren, 2005; Beals and Hill, 2006; Nattiv et al., 2007; Nichols et al., 2007; O'Donnell et al., 2009; Logue et al., 2018; Estienne et al., 2021). The studies which examined elite athletes found that menstrual disturbances were more prevalent among athletes compared to control populations (Wade et al., 1996; Torstveit and Sundgot-Borgen, 2005; Nattiv et al., 2007). The studies which compared athletes in lean-build sports to those in non-lean-build sports found that menstrual disturbances were more prevalent among athletes in lean-build sports than those in non-lean-build sports (Beals, 2002; Goodman and Warren, 2005; Nichols et al., 2007; Estienne et al., 2021). These studies concluded that athletes participating in sports that emphasize leanness report menstrual disturbances at a higher rate than athletes participating in less weight-sensitive sports. Additionally, one study reported that beginning high-intensity training at a young age is related to delayed onset of menarche (O'Donnell et al., 2009).

**Table 1:** Prior research examining menstrual dysfunction in athletes*.

**Study**	**Sample**	**Criteria for amenorrhea classification**	**Results**	**Conclusion**
Beals, 2002	23 elite volleyball players	Not stated	Amenorrhea: 17% Oligomenorrhea: 13% Irregular cycles: 48%	Elite adolescent volleyball players are at risk for MD (worse during competitive season) and have energy and nutrient intakes that placed them at risk for nutritional deficiencies and compromised performance.
Cobb et al., 2003	91 competitive female distance runners - Age: 18–26 years	Amenorrhea: fewer than 4 cycles in the past year Oligomenorrhea: 4–9 cycles in the past year	Amenorrhea: 10% Oligomenorrhea: 26%	In young competitive female distance runners disordered eating was strongly related to MI and MI was associated with BMD.
Torstveit and Sundgot-Borgen, 2005	669 Norwegian elite athletes and 607 controls - Total population of elite female athletes in Norway - Age: 13–39 years	Primary amenorrhea: absence of menarche by age 16 Secondary amenorrhea: absence of three or more consecutive menstrual cycles after menarche Oligomenorrhea: menstrual cycles of 35 days or more	Primary amenorrhea: - Athletes-7.3% - Controls-2.0% Secondary amenorrhea: - Athletes in leanness sports-24.8% - Non-leanness sports-13.1%	Age at menarche was later and prevalence of primary amenorrhea was higher in elite athletes than controls. A higher percentage of athletes competing in sports that emphasize thinness reported MD than athletes in less weight sensitive sports.
Castelo-Branco et al., 2006	38 adolescent dancers engaged in high intensity training and 77 controls - Age: 12–18 years	Primary amenorrhea: absence of menarche by age 16 Secondary amenorrhea: absence of three or more consecutive menstrual cycles after menarche	Amenorrhea: - Athletes: 8% Oligomenorrhea: - Dancers-34% - Controls-14%	Early, high-intensity training delayed onset of menarche.
Beals and Hill, 2006	112 US collegiate athletes - Lean-build: 65 - Non-lean-build: 47 - Age: avg 19.5 years	Delayed menarche: absence of menarche by age 16 Secondary amenorrhea: absence of three or more consecutive menstrual cycles after menarche Menstrual irregularity: fewer than 12 cycles in past 12 months, greater than 10-day variation in cycle	Menstrual dysfunction: 25% of athletes - Lean -build: 32% - Non-lean build: 17% Delayed menarche: 9% of athletes Secondary amenorrhea: 21%, greater prevalence among lean-build	Amenorrhea was more common among athletes in lean-build sports. MI increased for both lean-build and non-lean-build sport athletes during the competitive season.
Nichols et al., 2007	423 high school athletes - Lean-build: 146 - Non-lean-build: 277	Primary amenorrhea: absence of menarche by age 15 Secondary amenorrhea: absence of three or more consecutive menstrual cycles after menarche Oligomenorrhea: menstrual cycles of <21 or >35 days	Menstrual irregularity: 20.1% - Lean-build: 26.7% - Non-lean-build: 16.6% Primary amenorrhea: 0.5% Secondary amenorrhea: 1.7% Oligomenorrhea: 18%	Prevalence of MI was higher in lean-build athletes than non-lean-build athletes. Athletes with DE were over two times as likely to report MI than athletes without DE.
Myrick et al., 2014	95 Division I collegiate athletes	Primary amenorrhea: absence of menarche by age 15 Secondary amenorrhea: absence of three or more consecutive menstrual cycles after menarche, no OC use Oligomenorrhea: <10 cycles in past 12 months, no OC use	Primary amenorrhea: 9.5% Secondary amenorrhea: 17.9% Oligomenorrhea: 35.8%	Athletes were at risk for MD. MD was perceived as common and normal in the female athlete. Prevalence of this perception was especially common in lean-build sports.
Rost et al., 2014	149 Swedish elite athletics (track and field) athletes	Not stated	Amenorrhea: 25%	Swedish female athletics athletes reported high prevalence of amenorrhea compared to normal population. More runners reported amenorrhea than athletes in throwing and jumping events.
Ravi et al., 2021	178 Finnish adolescent athletes and 105 non-athletes - Adolescence (14–16 years) - Young adult (18–20 years) 52 Finnish young adult athletes and 159 non-athletes	Primary amenorrhea: absence of menarche by age 15 Secondary amenorrhea: absence of three or more consecutive menstrual cycles after menarche Oligomenorrhea: menstrual cycles of 35 days or < 10 cycles per year	Current primary amenorrhea: - Adolescence: 4.7% - non-athletes: 0% Current MD - Young adult athletes: 38.7% - Non-athletes: 5.6%	Primary amenorrhea and MD were more common among athletes than non-athletes.

* MD, Menstrual dysfunction; MI, menstrual irregularity; DE, Disordered eating; OC, Oral contraceptive.

Menstrual dysfunction in athletes may increase susceptibility to injury, and/or lead to long-term health complications, such as endothelial dysfunction and low bone mineral density (De Souza and Williams, 2004; Beals and Hill, 2006; Warren and Chua, 2008; Pollock et al., 2010; Myrick et al., 2014; Armento et al., 2021). Research has indicated that the longer the duration of menstrual disturbance, the larger the bone mineral deficit at non-weight bearing sites (De Souza and Williams, 2004; Armento et al., 2021). Further, later in life when bone loss occurs more rapidly, women may already have significantly depleted bone mineral stores, placing them at higher risk for osteoporosis (Beals and Hill, 2006; Pollock et al., 2010; Myrick et al., 2014). To mitigate these health concerns, when athletes miss a period or recognize they are experiencing an irregular menstrual cycle patterning, it should serve as an obvious warning sign of potential RED-S, and athletes should seek immediate help from a health professional. Unfortunately, an absence of menstruation is often rejoiced and normalized within the sports environment (Goodman and Warren, 2005; Armento et al., 2021).

Sport climbing is highly competitive. Research indicates that elite climbers are generally shorter, leaner, and lighter than non-climbing athletes and they exhibit anthropometric profiles similar to gymnasts, ballet dancers, and long-distance runners (Watts et al., 2003; Sheel, 2004; Giles et al., 2006). Athletes competing in these types of sports have displayed an increased prevalence of disordered eating and chronically low body weight (Sundgot-Borgen and Garthe, 2011). Studies assessing eating behavior or nutrient intake among climbers have found disordered eating (Joubert et al., 2020), energy restriction (Zapf et al., 2001; Michael et al., 2019; Sas-Nowosielski and Wycislik, 2019), and iron deficiencies (Gibson-Smith et al., 2020). Some research indicates no correlation of low BMI and high climbing performance (Grønhaug, 2019), yet anecdotal evidence points to a climbing culture that values thinness and often encourages athletes to minimize weight through restrictive eating habits (Leslie-Wujastyk, 2019; Lucas, 2021). This drive for a high strength-to-weight ratio may cause athletes to maintain a state of chronic LEA, which may subsequently lead to menstrual disturbance. The prevalence of amenorrhea in climbers is currently unknown. Thus, the aim of this novel study was to determine the prevalence of amenorrhea among elite competitive climbers.

## Materials and methods

### Survey tool

An online survey (Qualtrics XM 2021, Provo, UT) was administered to all female climbing competitors registered with the International Federation of Sport Climbing (IFSC). The survey was approved by the ethics committee at Northern Michigan University (IRB#HS21-1208) and distributed by the IFSC to 1,500 IFSC members *via* email. There were 57 Member Federations within the IFSC at the time this manuscript was submitted and the IFSC may add/delete Federations annually (i.e., Croatia and Puerto Rico were added for 2022).

The survey was open from June 21 through August 11, 2021. The survey contained 33 questions divided into five sections: (1) Informed consent, (2) Demographics, (3) Climbing experience, training volume, climbing discipline, and injury history, (4) Menstrual history, contraceptive use, and diagnosed medical conditions, and (5) Self-reported weight and weight changes, self-reported height, eating behavior and eating disorder history (see the Supplementary materials for the survey's complete question set).

### Participants

Participation was optional, responses were collected anonymously without collecting personal identification. Inclusion criteria were self-identified and as follows: competition category identified as female, English was either their primary or secondary language, an IFSC registered athlete, and not pregnant or lactating within the past 12 months.

### Menstrual cycle status

Climbers who answered all menstrual cycle questions were categorized as amenorrhoeic if they answered this way to all of the following criteria defining amenorrhea (De Souza and Williams, 2004; Torstveit and Sundgot-Borgen, 2005):

- No menstrual bleeding for at least three consecutive months in the past 12 months
- No menstrual bleeding in the past 90 days
- Had three or fewer menstrual cycles in the last 365 days
- No menstrual cycle during entire life
- Self-characterize menstrual cycles as irregular

Eumenorrhea, also listed as non-amenorrhea, was used to categorize climbers by their answers according to the following criteria (Torstveit and Sundgot-Borgen, 2005; De Souza et al., 2010):

- Had 10 or more menstrual cycles (period comes every 25–35 days consistently) in the past 12 months
- Had menstrual bleeding in the past 90 days
- Had eight or more menstrual cycles in the last 365 days
- Never longer than 35 days in between cycles
- Self-characterize menstrual cycles as regular

When answers were not congruent about menstrual cycle status or did not meet either criteria, climbers were categorized as unsure. We asked the same question in two different ways to attempt to check for consistent answers (one year = 12 months and 365 days).

To help determine how regular the participants felt that their cycles were, a slider bar with a drag and drop function with choices of 0.1 to 5.0 was employed to self-characterize their cycles within the past 12 months as regular (0.1 to 1.5) or irregular (3.5 to 5.0), and if they selected scores from 1.6 to 3.4 then these data did not contribute to the characterization of amenorrhea or eumenorrhea. Thirty-three climbers (29%) self-characterized their cycles as irregular.

### Statistics

Results were computed using IBM SPSS Statistics for Windows, version 28.0 (IBM Corp., Armond, N.Y., USA). The data are presented using descriptive statistical summary measures (number of athletes), percentages, or means ± standard deviations.

## Results

### Survey tool results

During the time of inclusion, 229 attempts to enter the survey were recorded and 147 participants met the inclusion criteria and answered at least 6 questions. A total of 114 female climbers completed the majority of the climbing, eating behavior and menstrual cycle related questions, which were included in this publication.

### Participant results

As it is unknown how many of the possible participants actually received the email with the survey link due to a variety of reasons (i.e., inactive email accounts, unopened email, filtered to junk mail), a response rate is unknown. There were 114 complete responses (*N* = 114). All participants were IFSC registered female sport climbers who represented 30 different countries with their anthropometrics and climbing training and competition characteristics are reported in Table 2. The climbers mean age was 22.4 ± 4.8 years. The overall average BMI of the group (*n* = 110) was 20.7 ± 1.9 kg/m^2^. Three climbers in the eumenorrhea group did not report their height and one climber in the amenorrhea group did not report their weight, thus BMI could not be calculated for 4 climbers.

**Table 2:** Anthropometry and climber characteristics.

**Characteristic** **(*n* = 114, unless otherwise noted)**	**Mean ±SD or percentage of athletes**
Age, y (*n* = 112)	22.9 ± 5.0
Height, cm (*n* = 110)	164.4 ± 5.9
Weight, kg	56.1 ± 6.9
BMI, kg/m^2^ (*n* = 110)	20.7 ± 1.9
Competition country (*n* = 113) *30 countries were represented. The top 7 are indicated here. The remaining 23 countries each represented 4.4% or less of the data reported*	Austria: 11.5% United States: 9.7% Germany: 8.8% Switzerland: 6.2% Canada: 6.2% India: 6.2% Great Britain: 5.3%
Age at first climbing competition, y (*n* = 113)	12.9 ± 5.1
Financial sponsorship received in past 6 months	Yes: 45% No: 55%
Current climbing competition category *could select all that apply*	Speed: 14% Lead: 35% Boulder: 53% Combined: 10% Have not competed in past year: 39%
Days trained per week, d	5.2 ± 1.7
Hours trained per train day, h (*n* = 105)	3.4 ± 1.2

Nine athletes reported that they were not currently training, but those that were training averaged 3.4 ± 1.2 h per training day and 5.2 ± 1.7 days per week. Of the athletes that indicated that they had more than one training session within the same day, 17.5% had double trainings one day per week, 27.2% had double trainings two days per week, 9.2% had double trainings three days per week, 7.0% had double trainings four days per week, and 2.6% indicated that five days a week they had double training sessions within the same day.

In the past 6 months, 39% of the athletes had not competed. Of those who had, 61% competed in more than one discipline (speed, lead, boulder), and 10% had competed in a combined event (scores tallied in two or more disciplines within the same competition). Additionally, 45% of the athletes received financial sponsorship within the past 6 months.

Table 3 reflects body weight manipulation and eating characteristics of the athletes. Nearly half of the athletes reported that they attempt to change their body weight for competition through various methods including reduced food intake or increased training. Only 17% do not consciously restrict food intake while 23% report daily restriction of food intake. Additionally, of the athletes classified as eumenorrheic, 13.4% revealed they currently struggle with an eating disorder, while 22.2% of the athletes classified as amenorrhoeic revealed they currently struggle with an eating disorder.

**Table 3:** Body weight manipulation and eating characteristics.

**Characteristic** **(*n* = 114, unless otherwise noted)**	**Percentage of athletes**
Attempt to change training weight (*n* = 105)	Yes: 23% No: 77%
Attempt to change competition weight (*n* = 105)	Yes: 45% No: 55%
Consciously restrict food intake	Never: 17% Rarely, a few times/yr: 13% Occasionally, maybe twice/mos: 21% Yes, at least 3–4 times/mos: 11% Yes, at least once per wk: 9% Yes, several days per wk: 6% Yes, daily: 23%
Injury during past 12 months	Yes: 53.5% No: 46.5%
Relationship with food (*n* = 59)	Sometimes struggle: 37% Like my relationship: 49% Dislike my relationship: 14%
Currently struggle with this eating disorder (yes to any; *n* = 17) *could select all that apply*	Anorexia nervosa: 2% Bulimia nervosa: 2% Binge eating disorder: 3% Orthorexia nervosa: 1% Night eating syndrome: 3% Avoidant/restrictive food intake disorder: 5% Eating disorder not otherwise specified: 3% Have disordered eating patterns: 25% Never had any of the above: 64% Unsure: 5%
At any time, diagnosed with or believed to have had this eating disorder (yes to any; *n* = 32) *could select all that apply*	Anorexia nervosa: 14% Bulimia nervosa: 6% Binge eating disorder: 8% Orthorexia nervosa: 2% Night eating syndrome: 3% Avoidant/restrictive food intake disorder: 8% Eating disorder not otherwise specified: 8% Have disordered eating patterns: 26% Never had any of the above: 53% Unsure: 6%

### Menstrual cycle status results

#### Athletes with amenorrhea

Within the past 90 days, 13.2% (*n* = 15) had not had a menstrual cycle and 14% (*n* = 16) had no menstrual cycles for 3 consecutive months or more during the past 12 months, and 9.7% (*n* = 11) had 3 or fewer menstrual cycles in the last 365 days. One athlete (21 y) indicated they had never had a menstrual cycle, which was confirmed with a consistent, “never menstruated” response for all menstrual cycle questions.

By consistently answering all questions indicative of an amenorrhea diagnosis, the prevalence of current amenorrhea (past 12 months) in these IFSC competitive climbers was 15.8% (*n* = 18), one climber with primary amenorrhea and 17 with secondary amenorrhea. Table 4 displays the menstrual cycle characteristics of the athletes. Their average BMI was 19.9 ± 2.4 kg/m^2^. Within the past 12 months, 1 participant with amenorrhea used a hormonal implant, indicated she used it for birth control and to reduce bleeding, and had been diagnosed with or treated for polycystic ovary syndrome (PCOS). One other athlete with amenorrhea used oral contraceptives for birth control reasons only.

**Table 4:** Menstrual cycle characteristics.

**Characteristic** **(*n* = 114, unless otherwise noted)**	**Percentage of athletes**
Age at menarche, y	11 or younger: 8% 12–14: 61% 15–16: 24% 17+: 4% Never menstruated: 1% Don't remember: 2%
Track period with calendar or app	Yes: 59% No: 30% Not menstruating: 10% Never menstruated: 1%
Perceived regularity of menstrual cycle (*n* = 102)	Regular: 48% Irregular: 31%
Menstrual Status Category	Primary Amenorrhea: 1% Secondary Amenorrhea: 15% Eumenorrhea: 72% Unsure: 12%
Used oral contraceptives or received hormonal treatment/contraceptives in past 12 months *could select all that apply*	Yes, oral contraceptives: 14% Yes, hormonal treatment (included medical treatment and/or hormonal intrauterine device-IUD): 6% Yes, hormonal contraceptives: 10% None of these were used during the past 12 months: 73% Yes, hormonal ring: 3% Yes, hormonal coil: 3% Yes, hormonal implant: 4%
Reason for oral or hormonal contraceptives/treatment *could select all that apply*	Birth control: 23% Reduce menstrual pain: 10% Reduce bleeding: 10.5% Regulate in relation to performance: 10.5% Otherwise menstruation stops: 2% None of the options explain the reason: 3%
Experienced changes to cycle with increased training intensity, frequency or duration	Yes: 32.5% No: 36% Unsure: 30%
Changes to cycle due to increased training *could select all that apply*	I don't bleed at all: 15% I bleed less over the same number of days: 22% I bleed fewer days: 15% I bleed more over the same number of days: 4% I bleed more days: 4% No changes happen to my menstrual cycle when my training increases: 4% I am unsure: 19%
Experienced changes to cycle with restricted or reduced food intake	Yes: 22% No: 49% Unsure: 29%
Changes to cycle due to restricted or reduced food intake *could select all that apply*	I don't bleed at all: 13% I bleed less over the same number of days: 10.5% I bleed fewer days: 11% I bleed more over the same number of days: 1% I bleed more days: 1% No changes happen to my menstrual cycle when I restrict my food intake: 4% I am unsure: 19%

The number of climbers with amenorrhea also reported some characteristics that may have contributed to this menstrual status and are listed in Table 5. The use of oral contraception and/or hormonal treatment (11.1%), a low BMI (22.2%), increased training volume (33.3%), and/or restricting food (50%), or have an eating disorder (38.9%) may alter menses in athletes individually or when some or all of these issues coexist.

**Table 5:** Plausible causes of amenorrhea.

**Cause**	**Climbers with amenorrhea (*n* = 18) and potential contributing characteristics**
Primary amenorrhea	1
Oral or hormonal contraception/treatment use	2
Low BMI (IFSC critical margins <18.0 kg/m^2^)	4
Increased training volume report no bleeding at all	6
With food restriction reported no bleeding at all	9
Eating disorder	7
Medical reason (PCOS)	2

#### Athletes with eumenorrhea

The majority of participants were categorized with eumenorrhea, 72.0% (*n* = 82). During the past 12 months, 62.3% had 10 or more menstrual cycles (i.e., had a period every 25–35 days consistently) and 85.1% indicated that they had a period within the past 90 days. 61.4% had 8 or more menstrual cycles in the last 365 days and 43.0% indicated they had never had more than 35 days in between their cycles. Forty-nine athletes self-characterize their menstrual cycles as regular. Their average BMI was 20.8 ± 1.8 kg/m^2^. Twenty-three eumenorrheic athletes reported using a form of oral or hormonal contraception (13 oral, 3 hormone ring, 3 implant, 1 coil, and 3 non-descriptive) with the majority of these athletes (*n* = 18) using it for birth control purposes. However, 10 athletes with eumenorrhea declared they also used it to regulate their cycles in relation to performance.

#### Athletes unsure of menstrual status

An additional 12.3% (*n* = 14) of the athletes had answers to menstrual cycle questions that were not congruent or not known to the athlete and therefore, menstrual status could not be determined. For example, 20.5% of these athletes indicated that their cycles were somewhere in between regular and irregular on the menstrual cycle characterization scale, 17.5% were unaware of the number of cycles that had occurred in the past 365 days and 10.5% were unaware of the number of cycles that had occurred over the past 12 months. Thus, these participants were categorized as unsure. Their average BMI was 20.9 ± 2.1 kg/m^2^. Two of these athletes used oral contraceptives, 4 used hormonal treatment or contraception and all six selected birth control as a reason. In addition, 4 of these 6 athletes with unsure menstrual status also selected the reason for using it was to reduce menstrual pain and to reduce bleeding. One of these athletes selected four reasons for use: to regulate my cycle in relation to performances, birth control, to reduce pain and to reduce bleeding.

## Discussion

The purpose of this study was to determine the prevalence of amenorrhea among elite level competition sport climbers and, to the authors' knowledge, it was the first study to do so.

The overall prevalence of amenorrhea in our sample was 15.8%. When compared with other studies surveying elite athletes, our results are similar to volleyball players at 17% (Beals, 2002), lower than elite Norwegian athletes in leanness sports at 24.8% (Torstveit and Sundgot-Borgen, 2005), and lower than Swedish track and field athletes at 25% (Otis, 1992). Table 1 compares additional studies examining prevalence of menstrual dysfunction in athletes. Furthermore, one review estimated the prevalence of secondary amenorrhea in athletes at 3–66% compared with 2–5% in the general population (Sas-Nowosielski and Wycislik, 2019).

Similar to our findings, Cobb et al. (2003) reported that disordered eating was strongly correlated with menstrual disturbances in female distance runners (Cobb et al., 2003). Torstveit and Sundgot-Borgen (2005), Nichols et al. (2007), and Beals and Hill (2006) also noted that competing in a sport that emphasizes thinness, or a strength-to-weight characteristic, increased the prevalence of menstrual disturbances (Beals, 2002; Torstveit and Sundgot-Borgen, 2005; Nichols et al., 2007). In the present study, only 4 of the 18 climbers with amenorrhea had a low BMI, <18.0 kg/m^2^. The IFSC uses BMI critical margin values to help screen athletes at competitions that may have compromised health and during the time of this research (2021), the BMI critical margins were set at 18.0 kg/m^2^ for female competitors during the World Cup sanctioned events. BMI screenings were arranged at every Boulder and Lead semi-finals done by competition doctors or Medical Commission members. However, the consequences for athletes that fall below these BMI critical margins follow a fairly flexible process. National Climbing Federations with critical cases receive a letter from the IFSC Medical Commission requesting an explanation on the specific athlete(s) and then may also receive support from IFSC Medical Commission members. Only seven climbers of the 114 in the present study had BMI values at or below these critical margins, four of which had amenorrhea. The other 14 climbers with amenorrhea had higher BMI values, which supports that using BMI criteria alone is not indicative of menstrual function.

This suggests that BMI should not be the only parameter used to decide the health status, nutritional status, or competition status of a climber. A low BMI may indicate further health assessment in a climber, but a BMI above 18 kg/m^2^ does not suggest that the climber is ensured to be healthy. Another tool applied by the IFSC is the Mass Index, which considers a long torso or long legs by using a formula with the sitting height or leg length to help adjust for body type differences. We did not include this measure in the present study. Currently, the International Olympic Committee has a working group researching the current status of body composition assessment in health and performance and an update on their previous report (Sundgot-Borgen et al., 2013) is hopefully forthcoming.

The aim of this study was to assess prevalence of amenorrhea and describe plausible causes for such, and not to assess nutritional status nor correlate any data with climbing performance. However, it is useful to draw on other studies that examined anthropometric data in female climbers. Giles et al. (2021) found that there was no significant difference in their female climber cohort between advanced and elite climbers for height, body mass, BMI, or skinfold thickness (Giles et al., 2021). The BMI for their elite climbers was 21.2 ± 2.0 kg/m^2^, which is slightly higher than those with amenorrhea in our present study. This may be relevant for practitioners to understand BMI in context of the whole athlete.

Assessing full nutritional and medical status is recommended by the IFSC for all athletes, particularly those that may report altering calorie or food intake to control weight, disordered eating, or history of disordered eating. In our cohort, 44% with amenorrhea altered their calories/food intake in order to cut weight for competition and 22% reported they currently had an eating disorder and 39% reported yes to having had an ED at any time during their life. Among a few other reasons listed in Table 5, these energy related reasons are plausible causes of amenorrhea.

These issues are consistent within the RED-S framework, which places athletes at a higher risk for amenorrhea when weight or energy intake is inadequate to support training and normal body function. It is noted that the IFSC sets and revises standards for a yearly sports medical examination to help ensure the health of its licensed athletes. This recommended sports medicine examination includes a complete sports physical that includes the climber's weight, height, BMI, Mass Index, body fat, flexibility, spiroergometry, standard blood/urine lab tests, electrocardiography, echocardiography, vaccinations, psychological stability, and nutrition screening. Although all of these are recommended by the IFSC, it is often up to each National Federation to health screen their athletes which may vary in rigor.

Some of the limitations and strengths of the present research must be highlighted. This study was cross-sectional and data were collected using an online survey written in English only, which narrows the representation of all competitive elite climbers. The survey tool used to assess prevalence of current amenorrhea was not a validated instrument. It was modeled off the Low Energy Availability in Females Questionnaire (LEAF-Q) (Melin et al., 2014), but was slightly modified to better serve the purpose of our study and ensure relevance to our population (see the Supplementary materials for the survey's complete question set). Furthermore, medical professionals and experts researching amenorrhea in athletes were consulted and their advice guided our survey development.

The lack of control of whether or not the emails that were sent out actually were received and whether or not the decision of not taking part was an active decline or was a result of not opening or receiving the email is another issue that must be addressed. Perhaps construed as a low response rate if 1,500 female climbers are registered with the IFSC and only 7.6% of them responded to all pertinent questions. This methodology issue does occur with online based surveys and must be taken into consideration when the results are interpreted and used. This preliminary data suggests further investigations with more objective measures, such as multiple sample hormone testing over several months, are required to truly determine amenorrhea prevalence of any given population. The surveyed prevalence value helps us to learn that this population is still vulnerable to reproductive health issues and that 16% is not a value to ignore. Instead, we need to focus more attention on placing proper screening within each National Climbing Federation and reproductive health education to support this population.

In addition, the timing of the survey may have had an impact on the reported body weight because the survey was open during the 3 months while most respondents were in the midst of the international competitive season. Finally, some participants may have purposefully lowered their weight for competition and the self-reported data collected may not have reflected their usual body weight, although the survey did ask participants about whether or not they altered their training body weight vs. competition body weight and how they went about it.

A strength of the study is that all participants were international elite climbers. Performing research with a specific group of athletes makes the results easier to interpret and use to develop medical screening guidelines, education and injury prevention strategies specifically targeted to this population. To the authors' knowledge, menstrual disturbance research among athletes from multiple countries has never before been published.

This study has captured scientific evidence on female climbers competing at the elite level, which indeed supports that this population is susceptible to menstrual dysfunction, eating disorders and disordered eating patterns. Future research should include a more comprehensive examination of the causes of amenorrhea and menstrual irregularity in climbing populations, energy availability assessment in competitive climbers and its correlation with performance and health and injury risk. This will facilitate the development of medical screenings and protocols within competitive climbing organizations to ensure female athletes are safe for competition with their health protected. Also, it will help to shape a safer team atmosphere with proper eating behaviors that support athletic endeavors and reproductive function. With more scientific evidence in this area practitioners will be better equipped to educate athletes and coaches with evidence-based health recommendations.

## Data availability statement

The raw data supporting the conclusions of this article will be made available by the authors, without undue reservation.

## Ethics statement

This study involved human participants which was reviewed and approved by the Internal Review Board of Northern Michigan University. Written informed consent to participate in this study was provided by the participant and all minors' legal guardian/next of kin.

## Author contributions

LJ, AL, GG, and MM designed the study. LJ, AW, AL, GG, MM, VS, and EB designed the survey tool. LJ, VS, and EB collected the data. LJ and GG performed the data analyses. LJ, AW, GG, and MM wrote the manuscript. All authors read, contributed to, and approved the final manuscript.

## Funding

Publication fees for this work were supported by Northern Michigan University's College of Health Sciences and Professional Studies internal grant.

## Conflict of interest

Author MM is self-employed by Real Nutrition, LLC and serves on the USA Climbing Medical Committee. She assisted with the survey question development and is not currently associated with a university, but has specific expertise in climbing nutrition, eating disorders, and female health. The remaining authors declare that the research was conducted in the absence of any commercial or financial relationships that could be construed as a potential conflict of interest.

## Publisher's note

All claims expressed in this article are solely those of the authors and do not necessarily represent those of their affiliated organizations, or those of the publisher, the editors and the reviewers. Any product that may be evaluated in this article, or claim that may be made by its manufacturer, is not guaranteed or endorsed by the publisher.
