# Supplementary material for: Prevalence of amenorrhea in elite female competitive climbers
Source: Front Sports Act Living. 2022 Aug 10;4:895588. doi: 10.3389/fspor.2022.895588 (PMC9400828; doi:10.3389/fspor.2022.895588)
Supplement: Supplementary file 1 [file Data_Sheet_1.DOCX]

***2021 Research Survey (33 Questions) Examining Menstrual Health Among Elite Competitive Female Climbers***

**Demographics Questions**

Q1 My current age today is...

- 15 years or younger
- 16 years or older

Q1a My specific age in years is...

Q2 The category I compete in is...

- Men
- Women

Q3 English is my...

- first language
- second language
- I do not speak English

Q4 Are you currently or have you been pregnant or nursing **during the past 12 months**?

Q5 My current height in centimeters is...

Q6 My current body weight in kilograms is...

Q7 The country I represent at Sport Climbing competitions is...

**Climbing experience and competition weight questions**

|  |
| --- |

Q8 My age in years when I first competed in Sport Climbing was...

Q9 I have received financial sponsorship as a Sport Climbing athlete **within the past 6 months**.

- Yes
- No

|  |
| --- |

Q10 **Within the past 6 months**, I have competed in this Sport Climbing discipline (select all that apply)...

- Speed
- Lead
- Boulder
- Combined
- I have not competed in the past 6 months

Q11 I am currently training for my next competition for this climbing discipline (select all that apply)...

- Speed
- Lead
- Boulder
- Combined
- I am not currently training for a competition

Q11a How many days per week do you train?

- 1
- 2
- 3
- 4
- 5
- 6
- 7
- I am not currently training

Q11b How many hours per day do you train?

- less than 1 hour
- 1
- 2
- 3
- 4
- 5
- 6
- 7
- 8
- 9
- 10

Q11c How many of your weekly training days do you have 2 or more training sessions within the same day?

- none
- 1
- 2
- 3
- 4
- 5
- 6
- 7

Q12 Some climbers purposefully change their body weight for different phases of their training. As an example, some climbers may choose to perform the majority of their training at a heavier body weight (training weight) compared to when they compete in competitions (competition weight). 


Do you try to achieve a specific "training weight" for your body?

- Yes, I try to achieve a specific training weight
- No, I do not try to achieve a specific training weight

Q12a My training body weight is usually...

- heavier than my competition weight
- lighter than my competition weight

Q12b How do you try to achieve your "training weight"? (please select all that apply)

- I change my calorie/food intake
- I change my training/exercise
- I change the amount of water I drink
- I change my smoking habits
- I use dietary supplements
- I use pharmaceuticals/medicine
- None of the above fit my experience, please explain: ________________________________________________

Q13 Some climbers purposefully change their body weight for different phases of their training. As an example, some climbers may choose to perform the majority of their training at a heavier body weight (training weight) compared to when they compete in competitions (competition weight).


Do you try to achieve a specific “competition weight” for your body?

- Yes, I try to achieve a specific competition weight
- No, I do not try to achieve a specific competition weight

Q13a My competition body weight is usually...

- heavier than my training weight
- lighter than my training weight

Q13b How do you try to achieve your "competition weight"? (please select all that apply)

- I change my calorie/food intake
- I change my training/exercise
- I change the amount of water I drink
- I change my smoking habits
- I use dietary supplements
- I use pharmaceuticals/medicine
- With purging behaviors
- None of the above fit my experience, please explain:

Q14 Do you consciously try to restrict the overall food that you eat, whether you succeed or not?

- Yes, daily
- Yes, several times a week
- Yes, at least once a week
- Yes, at least 3-4 times a month
- Occasionally, maybe twice a month
- Rarely, a few times a year
- Never

**Climbing Injury questions**

Q15 Have you experienced an injury during the past 12 months?

- yes
- no

Q15a How many injuries have you experienced during the past 12 months?

- 1
- 2
- 3
- 4
- 5
- more than 5

Q15b Select acute (happened suddenly) or chronic/overuse (on-going issue) for each body part injured during the past 12 months. Please leave empty if the body part was not injured...

|  |  |
| --- | --- |
| Toe | ▼ Acute ... Chronic/Overuse |
| foot/ankle | ▼ Acute ... Chronic/Overuse |
| calf | ▼ Acute ... Chronic/Overuse |
| knee | ▼ Acute ... Chronic/Overuse |
| thigh | ▼ Acute ... Chronic/Overuse |
| hip | ▼ Acute ... Chronic/Overuse |
| lower back | ▼ Acute ... Chronic/Overuse |
| shoulder | ▼ Acute ... Chronic/Overuse |
| elbow | ▼ Acute ... Chronic/Overuse |
| wrist | ▼ Acute ... Chronic/Overuse |
| finger(s) | ▼ Acute ... Chronic/Overuse |
| neck | ▼ Acute ... Chronic/Overuse |
| head | ▼ Acute ... Chronic/Overuse |

Q15c Did you seek a health professional for treatment or care for your injury or injuries?

- yes (for one or more)
- no
- I am a health professional

**Menstrual history, disturbances, oral contraceptives questions**

Women’s health issues are often neglected in sports-related research. Your participation in this survey will help to protect female athletes and we thank you for being willing to honestly answer the next set of questions about your menstrual cycle.

A menstrual cycle is defined as the length of time from when you begin bleeding and ends the next time you begin to bleed (an average length of a menstrual cycle is 28 days). A period, or time of menses, is defined as the time when you experience bleeding (average length of a period is 6 days). If you use a calendar or phone app to help you keep track of your menstrual cycle, please have this information handy while completing the next few questions.

Q16 Do you mark on a calendar or use a menstrual cycle or period tracking device and/or phone app?

- Yes, I track using a calendar, device or app
- No, I do not track the timing of my menstrual cycle, but I do have menstrual cycles
- I do not currently menstruate so I have no need to track cycles
- I have never menstruated so I have no need to track cycles

Q17 How old were you when you first menstruated?

- 11 years old or younger
- 12-14 years old
- 15-16 years old
- 17 years or older
- I don’t remember
- I have never menstruated

Q18 Do you currently weigh more or less than you did when you first menstruated?

- I weigh more now than I did when I first started
- I weigh less now than I did when I first started
- I weigh the same
- I don't know
- I have never menstruated

Q19 Have you ever had any of the following surgical procedures (check all that apply):

- Hysterectomy
- Ovarian cyst removed
- Oophorectomy (removal of one or both ovaries)
- Tubal ligation
- Other gynecological procedure not listed here
- I have never experienced any of these surgical procedures

Q20 Did your first menstrual cycle come naturally by itself?

- yes
- no
- I don't remember
- I have never menstruated

Q20a
What kind of treatment was used to start your first menstrual cycle?

- Hormonal treatment (patch, coil, ring, implant)
- Oral contraceptives
- none of the treatment options listed were used to start my menstrual cycle
- I am unsure

Q21
How would you characterize the monthly regularity of your menstrual cycles **during the past 12 months**?

|  | Regular | Not regular |
| --- | --- | --- |

|  | 0 | 1 | 1.5 | 2 | 2.5 | 3 | 3.5 | 4 | 4.5 | 5 |
| --- | --- | --- | --- | --- | --- | --- | --- | --- | --- | --- |

| slide the bar | 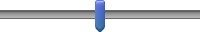 |
| --- | --- |

Q22 **During the past 12 months**, this is true about my menstrual cycles...

- I have had 10 or more menstrual cycles (I get my period every 25-38 days consistently)
- I have not had menstrual cycles for 3 consecutive months or more
- I am unaware of the number of menstrual cycles I have had during the past 12 months
- My menstrual cycles do not fit any of these choices

22a **During the past 365 days**, I have had _____ menstrual cycles. (fill in the blank or check below)

- I am unaware of the number of menstrual cycles I have had during the past 12 months

Q22b Has your menstrual cycle length ever been longer than 35 days?

- yes
- no
- I'm not sure

Q22c Have you had a period **within the last 90 days**?

- yes
- no
- I'm not sure

Q23
**During the past 12 months**, have you used oral contraceptives or received hormonal treatment or used other hormonal contraceptives? (check all that apply)

- yes, oral contraceptives
- yes, hormonal treatment (includes medical treatment and/or hormonal intrauterine device - IUD)
- yes, hormonal contraceptives
- none of these were used during the past 12 months

Q24
**During the past 12 months**, have you used any of the following treatments and/or medications? (check all that apply)

- Oral contraceptives
- Hormonal patches
- Hormonal ring
- Hormonal coil
- Hormonal implant
- Steroid medication (cortisone shots, prednisolone, etc)
- These choices don't exactly describe the type of oral contraceptives I’ve used or hormonal treatments I’ve received during the past 12 months
- I have NOT used any of the above or any other oral contraceptives, hormonal treatment or steroid medications during the past 12 months

Q25 **During the past 12 months**, please indicate the reason you used oral or hormonal contraceptives...(select all that apply)

- Birth control
- Reduce menstrual pain
- Reduce bleeding
- Regulate my menstrual cycle in relation to performances, etc
- Otherwise menstruation stops
- None of these options describe why I use oral contraceptives

Q26
**During the past 12 months**, did you experience changes in your menstrual cycle when you increased your training intensity, frequency, or duration?

- yes
- no
- I am unsure
- I have never had a menstrual cycle

Q26a Which changes in your menstrual cycle do you experience due to your increased training (check all that apply)...

- I don’t bleed at all
- I bleed less over the same number of days
- I bleed fewer days
- I bleed more over the same number of days
- I bleed more days
- no changes happen to my menstrual cycle when my training increases
- I am unsure

Q27
**During the past 12 months**, did you experience changes in your menstrual cycle if you restricted or reduced the overall amount of food that you ate?

- yes
- no
- I am unsure
- I have never had a menstrual cycle

Q27a Which changes in your menstrual cycle do you experience due to your restricted or reduced overall food consumed (check all that apply)...

- I don’t bleed at all
- I bleed less over the same number of days
- I bleed fewer days
- I bleed more over the same number of days
- I bleed more days
- no changes happen to my menstrual cycle when my training increases
- I am unsure

Q28
Has your body weight changed more than 3 kilograms **during the past 12 months**?

- yes
- no
- I am unsure

Q28a My body weight...

- increased more than 3 kgs during the past 12 months
- decreased more than 3 kgs during the past 12 months
- fluctuated both up and down by more than 3 kgs during the past 12 months

Q28b **During the past 12 months**, did you experience changes in your menstrual cycle when your weight changed?

- yes
- no
- I am unsure
- I've never had a menstrual cycle

Q28c Which changes in your menstrual cycle do you experience due to your body weight changing (check all that apply)...

- I don’t bleed at all
- I bleed less over the same number of days
- I bleed fewer days
- I bleed more over the same number of days
- I bleed more days
- no changes happen to my menstrual cycle when my body weight changes
- I am unsure

Q29
Have you ever intentionally prolonged your menstrual cycle to avoid having your period during Sport Climbing competitions?

- yes
- no

Q30 How would you characterize your relationship with food?

- I like my relationship with food
- I sometimes struggle with my relationship with food
- I don't like my relationship with food

Q31
I **currently struggle** with the following (select all that apply)...

- Anorexia nervosa
- Bulimia nervosa
- Binge eating disorder
- Orthorexia nervosa
- Night eating syndrome
- Avoidant/Restrictive Food Intake Disorder (ARFID)
- Eating disorder not otherwise specified (EDNOS)
- I have disordered eating patterns
- I don't have any of the above issues currently
- I am unsure

Q32
At **any time during your life** have you ever been diagnosed with or believe you have had any of the following eating disorders?  (select all that apply)

- Anorexia nervosa
- Bulimia nervosa
- Binge eating disorder
- Orthorexia nervosa
- Night eating syndrome
- Avoidant/Restrictive Food Intake Disorder (ARFID)
- Eating disorder not otherwise specified (EDNOS)
- I have disordered eating patterns
- I have never had any of the above issues
- I am unsure

Q33 I have been diagnosed or treated for this/these condition(s)... (check all that apply)

- Polycystic Ovary Syndrome (PCOS)
- Menorrhagia
- Uterine fibrosis
- Endometriosis
- Pelvic inflammatory disease
- Any type of gynecological cancer
- I have never been diagnosed with or treated for any of these conditions
